# Important to County Medical Societies

**Published:** 1889-09

**Authors:** 


### IMPORTANT TO COUNTY MEDICAL SOCIETIES.

We beg to call the attention of secretaries of County and District Medical Societies in Texas to the following ordinance, passed by the Texas State Medical Association at its eighth annual session,—an important matter, which has, as a general thing, heretofore been entirely neglected. Were the requirements of the ordinance complied with, it would aid largely in the organization of the profession, by enabling the Secretary to put into the hands of every physician the literature of the Association, invitations to attend meetings, etc., and to preserve valuable statistics:

"*Resolved*, That County Medical Societies be requested to furnish [to the Secretary of the T. S. Med. Ass'n] a list of physicians in the county, as well as of members; to give population of county, number of births, deaths, cause of death, age at death, sex and race; diseases that have prevailed; localities, with a description of the latter, in order to ascertain, if possible, the causes of disease; also an abstract of contents of papers read, and of discussions, surgical operations and results."

We call upon the secretaries of the several societies in affiliation with the State Association to furnish us, at once, with (1) an account of the organization of their respective societies; (2) officers for the present year, and list of members; (3) a list of physicians in their respective counties, with post-office addresses.

Societies which comply at once with the above request will receive due credit, and a copy of the Transactions for '89 for the society library.

In this connection, we beg to point out for the information of those members of county societies and others who may hereafter represent their respective societies and counties on the Nominating Committee, that for some years past the Nominating Committee have not fully complied with the by-laws in the election of officers. The secretary, as well as the chairman, of every section, must be elected by the Nominating Committee, and at the same time, instead of being appointed by the chairman; which is seldom done in time to have the names of secretaries appear in the Transactions. This is very important. [See by-laws under head of "Sections," page 276-7, Transactions 1889.] And, with regard to the Sections of State Medicine and Public Hygiene, the by-laws say this Section "shall be composed of *one member from each county, representing, as far as practicable, the county Board of Health*, the officers of this section to be also designated by the Nominating Committee."

Under the head of "Standing Committees," the by-laws also provide for a "Committee on Texas State Medical Necrology." "This committee to consist also of one member from each county represented in the Association." The duties of said committee are defined in same paragraph.

Recently the Nominating Committee have entirely neglected this important appointment. At last meeting (San Antonio) the Secretary called attention to the omission from report of Nominating Committee, whereupon the President appointed *two* members, Drs. A. W. Pope and B. W. Bristow. Some record of our departed brethren should be made and preserved in the archives of the Association.

